# Instruments and agents of control: smallpox in the Río de la Plata at the beginning of the nineteenth century

**DOI:** 10.1590/S0104-59702026000100001en

**Published:** 2026-02-23

**Authors:** María Silvia Di Liscia

**Affiliations:** i Full professor, Instituto de Estudios Históricos y Sociales de La Pampa/Universidad Nacional de La Pampa. Santa Rosa – La Pampa – Argentina silviadiliscia@gmail.com

**Keywords:** Smallpox, Río de la Plata, Nineteenth century, Immunization

## Abstract

Different systems for controlling smallpox in the early nineteenth century are investigated, considering the actions of different agents, mechanisms, and strategies involved in its organization, manufacture, and distribution. This dangerous disease emerged in the Americas following European colonization. Immunization practices arrived in the Viceroyalty of the Río de la Plata in 1805, coordinated first by the Protomedicato and later through the Conservatory of Vaccines. The economic hardship and wars of the fledging state hindered the inoculation of human and cowpox viruses. Humans were used as viral reservoirs, yet the epidemics remained uncontrolled. Replication of European experiments to obtain similar viruses failed to provide a safe product.

## Changes in smallpox and its prevention

As a disease now extinct, smallpox was a significant threat for centuries due to a lack of immunity among people, leading to severe consequences for the health of survivors. The *variola* virus is transmitted from person to person through the air. The disease begins with fever and a full-body rash, which can result in permanent scars and, in some cases, blindness. Unlike other diseases, smallpox was widely preventable through methods such as arm-to-arm inoculation and variolation.^1^ However, both methods, in the nineteenth century, were unsafe, risky, and could even be fatal, especially for minors.

The term “vaccination” was established after 1881, when Louis Pasteur first used it to name his rabies vaccine in homage to Edward Jenner. It was used to refer to all artificial products introduced into the bodies of humans or animals to generate an immunological and preventative response to a specific disease (Moulin, 2010). The smallpox vaccine is considered one of the “transferable technologies” that saved millions of lives worldwide and enabled the eradication of the disease (Altenbaugh, 2018, p.4).

The extensive literature on smallpox and its global vaccination response cannot be fully examined here (Holmberg et al., 2017; Blume, 2017). However, it is essential to consider recent contributions that explore the relationships between the information on prevention practices and strategies dating back to the late eighteenth century (Few, 2010; Bhattacharya, Brimnes, 2010). In the context of what is now the Argentine Republic, there are updated studies on the impact of smallpox and its control within the national territory (Jiménez, Alioto, 2017; Di Liscia, 2023).

Our objective is to explore the early decades of the nineteenth century, a time when the American landscape was transformed by the general process of independence, leading up to the political reorganization attempts of the mid-nineteenth century. As immunization practices were practically the only public health activity, our study also focuses on the profiles of key figures such as Chief Physician Miguel O’Gorman, Dean Saturnino Segurola, and Naturalist Francisco Xavier Muñiz. Also, we consider other important people linked to inoculation practices, such as entrepreneurs benefiting from these procedures and children who were used as reservoirs. Our research draws on a variety of chronicles, reports, official records, and press material from the period, and published and unpublished texts and theses by contemporary physicians.

## The protagonists

### During the colonization

Much of the current territory now called Argentina was not governed by the Spanish Empire. Many indigenous peoples settled there and moved across a diverse and resource-rich area from north to south. Despite being separated by borders and experiencing periods of conflict, the Spanish and their Creole descendants, mestizos, Black people, and Indigenous peoples shared a collective existence, often intermingling in times of peace (Di Meglio, 2012).

In 1776, the Viceroyalty of the Río de la Plata was established. Its capital, the port of Buenos Aires, was founded in the sixteenth century and was then a small town. To the west lay the lands inhabited by various indigenous ethnic groups. The population was unevenly spread across a vast and poorly connected area including Salta, Tucumán, and Jujuy in the north; the Cuyo region, corresponding to the provinces of Mendoza, San Juan, and San Luis; and the central region, with Córdoba and Santiago del Estero, as well as the Litoral provinces. In these territories, religious orders, relatively isolated from royal authority, also held significant influence. As in other regions, the metropolis consisted of cities governed by councils (Maeder, 2018).

Smallpox was introduced to the Americas during the period of conquest and colonization. The disease had a high morbidity and mortality rate among indigenous populations throughout the continent as their immune systems lacked the “memory” needed to combat illnesses that Europeans and Africans had experienced for centuries (McNeill, 1976). In the Viceroyalty, the disease emerged in the seventeenth century, with outbreaks in Buenos Aires and possibly throughout the rest of the country.^2^

The success of the Spanish occupation relied on the use of indigenous people as servants and forced laborers, prompting conquistadors and missionaries to implement inoculation. This practice took place at the end of the eighteenth century among the Guaraní people of the northeastern missions, within the Jesuit empire, well before the arrival of Francisco Balmis’s expedition to the continent (Jiménez, Aliotto, 2017). In these settlements, deadly smallpox epidemics occurred in 1732, 1733, 1738, and 1764, and the priests inoculated 7,414 indigenous individuals (Penna, 1885). This approach was the only available strategy during the epidemics and consisted of taking scabs containing the smallpox virus from a survivor and applying them to another person for immunization. While this method offered some degree of protection, its effectiveness was limited since healthy individuals could still contract the disease, albeit in a milder form. Additionally, improper hygienic conditions or the donor’s pre-existing illnesses could lead to infections or the transmission of other diseases.

During the colonial period, many healthcare responsibilities, such as vaccination, were managed by the Royal Protomedicato. It emerged in Spain in the fifteenth century, and it was composed of technicians responsible for regulating the medical practice and teaching duties. A counterpart was established in the Americas in 1570 within the Viceroyalties of New Spain and Peru (Lanning, 1997). In 1779, Juan José Vértiz, the viceroy overseeing the Río de la Plata, requested the establishment of the Protomedicato, along with the Casa Cuna for foundlings (Gutiérrez, 1877; Babini, 1949). However, it wasn’t until 1798 that the Crown formally appointed the chief physician. Due to the prevalent inefficiencies in bureaucratic processes – a common complaint – the official designation was eventually carried out.

The Irish physician Miguel O’Gorman was in charge of the Protomedicato in the Río de la Plata from 1798 until its virtual dissolution in 1822. He graduated in Paris as a medical doctor, and he entered the Spanish service in 1766. He was sent to England to specifically study the variolation process, a method he later successfully applied, inoculating the entire Spanish Court (Ramírez Martín, 2022). O’Gorman’s situation in Buenos Aires was never easy, and even the monarch, Charles IV, had doubts about his appointment as a chief physician, given that he was considered an English spy (Damiani, 2020).

The quality and quantity of medical services in Buenos Aires were limited to a few hospitals, operated by religious orders for both men and women. Throughout the Río de la Plata region, there were only a few poor buildings, which were extremely precarious and suffered from notable shortcomings (Damiani, 2020). This situation is not surprising, considering that these facilities were far from sanitary and only the very poor people were treated. Individuals with certain financial resources had doctors and paid for expensive prescriptions in pharmacies and other drug supply centers, which the Protomedicato also had to control.

In 1799, the Protomedicato was composed of O’Gorman and six other officials, of whom only three held medical degrees. To “prevent empiricism in the Viceroyalty” (Gutiérrez, 1877, p.389), a Royal Decree authorized the creation of a medical chair, taught by O’Gorman and a surgeon (Agustín Fabre). However, in 1801, there were only nine students enrolled. This limited enrollment was due to a lack of resources. Future professionals did not have access to an amphitheater or chemistry laboratory and were required to produce their dissection instruments. The first graduates who were poorly trained were in 1806 (Gutiérrez, 1877).

In 1803, three doctors and 29 licensed physicians were qualified, with penalties for those unqualified (Gutiérrez, 1877). Authorities blamed the population for entrusting their health to quacks and fake doctors. However, during this period, medicine lacked scientific foundations, and the two specialists differed more in the exercise of medical authority than in experimental certainty (Di Liscia, 2002).^3^

Despite its limitations, information about the new immunization techniques to prevent smallpox was already accessible in Buenos Aires, largely because O’Gorman had firsthand knowledge from his travels and contacts in England. Unlike inoculation, where the live virus was introduced from a smallpox survivor into a healthy person, this new approach centered on the use of cowpox. This could be done through containers or vis human carriers. Although the former method was feasible, it was not widely recommended, as the resulting fluid dry proved ineffective. The latter method involved using people who have characteristics as carriers of the cowpox virus, who would transmit it to others with a lancet. Jenner even highlighted, with allusive drawings, the expected appearance of the pustule resulting from needle inoculation, and the waiting time; if this did not occur, the procedure would be deemed invalid (Jenner, 1802).

The beginning of variolation in the Río de la Plata can be traced back to the arrival of Portuguese businessman Antonio Machado Carvalho, who arrived in early August 1805 aboard a ship with inoculated slaves. He brought the “true vaccine” delivered by “two small black men” (Díaz, 1876, p.15; Caffarena Barcenilla, 2016). Once the ship arrived at the port of Buenos Aires, O’Gorman called upon the entire population to the Real Fortaleza (Royal Fortress). Between August 1 and 4, vaccinators Justo García Valdez and Silvio Gafarat inoculated 34 people. By August 9, thanks to several people who had received the “true vaccine,” the immunization extended to 200 more people (Díaz, 1876; Coni, 1878). Penna (1885) noted that among those inoculated were those from the Casa de Niños Expósitos (Foundling Home).

The use of the adjective “true” to describe the product extracted from children’s arms is because, at the time, there was a preventive measure against other products of limited effectiveness. Thus, no similar experiments related to the reproduction of cowpox as Jenner’s are cited since in both regions people were in contact with cattle. This situation may be due to medical studies being predominantly influenced by a Galenic paradigm, with little concern for veterinary issues.

In 1805, O’Gorman wrote an instructional document on the practice, which was published by the printing press of the Casa de los Niños Expósitos. This is a rarity, as it is the only one regarding medical matters in a wealth of official bureaucratic writings, such as edicts, royal decrees, guides, religious texts, and reports (Maeder, 2018). This document may have served as a model for others such as Saturnino Segurola, who began his activity in 1807. He was born in Buenos Aires in 1776 and passed away there in 1854. Segurola obtained a doctorate in theology in Chile. At the beginning of the nineteenth century, he focused on the control of smallpox, introducing the vaccine to Santiago in 1805. Although not a doctor, he was interested in various historical and cultural subjects, amassing a remarkable collection of maps, official documents, printed newspapers, sermons, memoirs, manuscripts, and objects donated to museums and libraries. He preserved them in repositories and archives until his death and later donated them to what is now the National Library.

In 1807, The Protomedicato, under the leadership of Justo García Valdez, made him responsible for the conservation of the vaccine. This may seem unimportant, but it was essential for the time because the Río de la Plata territory had recently been invaded by English frigates a few months earlier. This event compelled the popular organization of militias due to the Viceroy’s abandonment of Buenos Aires, an episode that demonstrated to the Creoles the limitations of imperial power, with implications for the subsequent process of independence (Goldman, 1998). Segurola played a fundamental role in preventing the loss of the “cow fluid” (Real Protomedicato..., 1940).

In 1805, slaves arrived carrying the virus (allegedly from cowpox) in their bodies. But how could this immunological transmission be used for others, through lancets and new applications that could preserve the “true vaccine,” in terms of time? Segurola was in charge of the procedure and likely used two methods at the same time: preservation the safe scabs in glass and using children, given his role as head of the Casa de Niños Expósitos. These theories remain unsubstantiated by documentary evidence, as the sources fail to elaborate on the techniques used – either due to their esoteric nature or because they were so apparent that there was nothing novel to report.

A year later, Segurola found himself defending against accusations regarding the type of product administered and the locations of the applications.

Who would believe that some sensible doctors, after asking me for Bacuna to honor their favorite people and publicly saying in the houses of this town that I was the only one who kept Bacuna and that if it were not for my desire it would have been extinguished in this town as well as in the entire Viceroyalty, would insist on not coming to my house because my liquid was of no use? (Note…, 1940, p.76).

With angry words, he accused his detractors of envy and of plotting a vile plan. According to him, their sick mind, instead of prioritizing the “public interest,” was motivated only by “their private interests.” At the same time, he invited the Protomedicato to

wash away this note of infamy that in some way may be transcendental to the members of this very respectable body, I find no other resource that your honor would be pleased to point out if it seems to you the most opportune day, calling the doctors you like to present several people vaccinated by my hand so that once the Smallpox is recognized, it may be revealed to the public, deceived by the means that prudence indicates to your honor the legitimacy of such a specific that I have administered to thousands of individuals of all ages (Note…, 1940, p.79).

In this document, the debate surrounding vaccination centers on the tension between individual rights and collective health benefits, not merely due to the right to refuse an artificial product aimed at preventing widespread contagion, but also because of its significance and financial implications. Segurola had vaccinated thousands for free, and those who profited from the vaccine, along with their fees, could have raised complaints about the product with the Protomedicato. The cleric proposed a solution: could it be proven, using the techniques of the time, that the immunized people were genuinely immune? What we do note is the limited institutional implementation of the measure, relying on a single person and their resources. The situation attempted to be somewhat modified during the subsequent government period, with relative success.

### Independence and beyond

In 1810, the news of the fall of the Central Board in Seville to the French invasion of the peninsula brought a new dynamic to the Río de la Plata region, where sectors were already keen to change and even to break away from the metropolitan government. Until 1820, what is broadly known as the “war of independence” unfolded throughout what is now Argentina, even involving indigenous groups. This military conflict began with expeditions launched by the Governing Board of Buenos Aires – to replace the viceroy – sent to different parts of the territory, with varying success. In subsequent years, the war situation became more complicated as other fronts emerged, involving the Litoral provinces, the Banda Oriental, and a powerful Portuguese offensive, while an army crossed the Andes to advance against the royalists from Chile and Peru (Di Meglio, 2007).

Simultaneously, a series of changes occurred in the government of what was then called the United Provinces of the South (Provincias Unidas del Sur). The militarization of the elite emerged in a context of enormous socioeconomic challenges, due to the impossibility of establishing a new political order, the transformations of government bodies, and a complicated international situation (Goldman, 1998). These issues undoubtedly impacted all institutions, including those dedicated to medical matters, which were already precariously developed.

In 1810, a curious document revealed that Miguel O’Gorman declared the transfer of the vaccine conservatory to the Provincial Governing Board. He also noted the efforts made since 1805 to maintain the conservatory and to provide vaccinations free of charge to other parts of the Viceroyalty. This was possible through two professors of medicine and an assistant at the Casa de Niños Expósitos and the Residence Hospital (Hospital de Residencia). Five years later, vaccination had been abandoned, and smallpox was spreading again. The reasons given were that those responsible for supplying and distributing the product had forgotten this obligation because they received no funds. In that environment, Segurola was noted as the only remaining successor. It was emphasized that some physicians spread notions contrary to vaccination among “less educated individuals,” promoting the institution to order them to “disimpress the public” under penalty of a fine (Real Protomedicato..., 1940, p.79).

In a tense political climate, the colonial authorities had attempted, with limited success, to variolate the virus, transferring that duty to the independent government. The Protomedicato continued, now under the control of Saturnino Segurola, who kept the preparation in glass containers within a specialized facility and attempted to disseminate it among the population. In 1813, a Medical Institute was established, which featured an anatomical amphitheater. Its main role was to train future doctors for military service (Gutiérrez, 1877). For this purpose, surgical knowledge was essential in addressing battlefield injuries, which could be fatal without a suitable medical staff. Also, this training aimed to mitigate the spread of smallpox through the inoculation of soldiers.

In 1811, Segurola documented the shipment of scrub between glass plates, indicating the recipient, Celedonio del Castillo. He advised him that, to use the scrub, they should “be ground down into 5 small or fine powders. Once this was verified, they were moistened with a few drops of common water to form a kind of fatty substance, which was then taken with a lancet and applied in the usual way” (Carta..., 1940a, p.82). This method was used until the end of the nineteenth century, even with variations in the binding substrate, and it is commonly referred to as a dry vaccine that required hydration to be effective. Its effectiveness was limited, leading to doubts about the reliability of the process in general (Di Liscia, 2023). Celedonio del Castillo was an important liaison between Buenos Aires and the Northeast during the wars of independence. The material was likely intended for the vaccination of soldiers and protect them from smallpox, but also for indigenous people, given that del Castillo served as the delegate governor of Apóstoles in Misiones, a historical area linked to the Guaraní Jesuit reductions (Codeseira del Castillo, 2012).

In 1813, with the support of Buenos Aires police authorities, Segurola published his famous regulation, which established a medical authority that was difficult to understand, considering its author was not the original author and required legal instruments that were difficult to implement. However, it was one of the few health-related regulations by the independent government. Its draft was based on the 1805 regulation, adjusting the reception, mandatory nature, and dissemination of vaccination. For example, it required neighborhood mayors, officials existing in the colony and who persisted until 1820, to compile a registry of both vaccinated and unvaccinated individuals, granting them police functions, as in Europe. Individuals were required to present a form confirming their vaccination, or they would risk getting a fine. The requirement was more demanding for families with young children and commanders of various army corps (Reglamento…, 1940).

The regulations indicated the intention to transport the vaccine beyond Buenos Aires, hinting at the difficulties, since there was no public space available to store and administer the vaccine: “For this operation, they will come, as they have done up to now, to my house, until the Police Judge, with the Honorable Council, arranges a house in the middle of the town where those interested can more conveniently attend” (Carta..., 1940b, p.87). This situation did not occur until well into the nineteenth century. Due to both laziness and organizational problems, as well as political difficulties, Segurola continued administering the vaccine almost privately in his residence on Puán street, as was later acknowledged in his biographies (La personalidad..., 1940).

The dean also responded to other requests from his political circle, such as the one from Manuel Belgrano to Thomas Sunter, the United States Minister Plenipotentiary to Brazil, who expressed a desire to “conduct experiments with the real vaccine” (Carta..., 1940c, p.88). In Rio de Janeiro in 1815, Belgrano was on other business and Sunter requested the vaccine, claiming that Segurola’s extensive knowledge of the vaccine had reached beyond the borders of the Río de la Plata. He also mentioned that “doctors assure us that it degenerates very quickly into smallpox” (Carta..., 1940c, p.88). Committed to scientific knowledge, Belgrano saw an error in such an assertion and asked Segurola to send the material to prove otherwise. However, such “degeneration” undoubtedly existed, as the virus could not survive without certain temperature and humidity conditions. If inoculated with live viruses, the disease could be transmitted to individuals who did not have it.

Almost immediately, the dean sent a package of vaccines to Brazil, stating with a certain arrogance that he had done so on more than one occasion to both the English and the Portuguese, who had not considered that the vaccine could transmit smallpox. This oversight had not occurred in Buenos Aires thanks to his intervention: “I have sent the vaccine countless times to that country, both for the English and the natives of the country, but as far as I understand, it has hardly had any purpose... because, as a result of not wanting to take the necessary measures to preserve it, the vaccine is often lost, so they resort to natural smallpox and ensure that the vaccine degenerates” (Carta..., 1940d, p.89).

Segurola gave the material to Sunter at no cost, who thanked him profusely (Carta…, 1940e, p.90). He attested to the vaccine’s efficacy, having personally tested it on one of his children (“I experienced its purity in one of my children”).^4^ Sunter said that he would come to Segurola’s defense, highlighting the quality of the product to the doctors of Rio de Janeiro: “I did the same as Dr. Jenner and… it is an honor to have advanced the usefulness of your noble discovery, which you have protected so selflessly for so many years” (Carta…, 1940e, p.91).

In the following years, Segurola continued to receive requests for vaccines from various locations, both to distribute to the families of his acquaintances and the general population. Between 1816 and 1821, Juan Martín de Pueyrredón requested the vaccine for Córdoba (Carta..., 1940f); Dámaso Antonio Larrañaga for Montevideo and the Banda Oriental (Carta..., 1940g); and Tomás Godoy Cruz for Mendoza. In Mendoza, the report indicated the population’s resistance to inoculation with ineffective products brought from Chile (Carta..., 1940h).

At that time, a new process was beginning in Buenos Aires, under the leadership of an elite group removed from centralization which initiated a series of liberal and autonomous policies. The town councils and their officials were abolished, leading to an expansion of the borders of Buenos Aires (Ternavassio, 1998). In 1822, in that same period of renewal, the Protomedicato was replaced by a Medical Tribunal (Sarmiento, 1885). Under the guidance of Bernardino Rivadavia, several key institutions linked to the medical and health situation emerged, such as the Sociedad de Beneficencia, in charge of hospices and hospitals; the Academia de Medicina, among other institutions of arts and sciences; and the University of Buenos Aires in 1821, which incorporated the previously created Medical Institute, although with only five chairs (Gutiérrez, 1877).

Since Segurola continued to be the custodian of the vaccine, he was ordered to send the “bovine virus” to the Carmen de Patagones fort (Order..., 1940). This and similar provisions reflect the government’s interest in implementing mandatory vaccination, with measures outlined in a circular addressed to the surgeons stationed at the border forts: Guardia del Monte, Chascomús, Magdalena, San Vicente, Luján, Pilar, Capilla del Señor, Cañada, and Areco. The system outlined decades earlier was reorganized with vaccine administrators and other officials who received a stipend of 45 pesos per month and were required to register those vaccinated. Transportation was even provided by horses belonging to the postmasters (Penna, 1885).

Segurola was accompanied by six other vaccinators, all authorized by an official publication, for this important task. In 1822, a report by Jenner outlined the vaccination methods, explained how the rash was produced, and provided other details. However, there was no mention of the process of obtaining the vaccine. We do not know how many people were vaccinated, but perhaps the quality of the product was not as desired, given that smallpox returned in 1825 (Penna, 1885).

Until 1829, Segurola performed a wide variety of tasks beyond those described above, both cultural, social, and political. He was a deputy in 1812, director of the Public Library, and in charge of the Casa de Niños Expósitos (Dr. Deán Saturnino..., 1940). However, with the rise of Juan Manuel de Rosas into the provincial scene in Buenos Aires, many of these activities came to an end. Notably, the care of the orphans at the Casa de Niños Expósitos was discontinued in 1838 due to budgetary reasons.

Rosas was governor from 1829 to 1852, when he was defeated by a coalition, ushering in another period that historiography frames as the beginning of the national order. The territory became known as the Argentine Confederation, with allied or warring provinces that maintained a degree of autonomy. During his administration, he implemented a policy of alliances with certain indigenous groups (the “friendly Indians”), who were located in Tapalqué and Azul, near the forts, and received rations. They also joined battalions to defeat other ethnic groups and extend the Buenos Aires border. Around 1830, a smallpox epidemic advanced in the countryside. The English consul in the Río de la Plata, Woodbine Parish (1958), stated that faced with the suffering of Rosas’s Indigenous allies, Rosas managed to convince them to inoculate the virus to be safe. We will not return to these aspects, which would imply the complexity of diplomatic relations between supposedly opposing groups, compared to the reality of alliances between indigenous people and whites, as we have examined them elsewhere (see Di Liscia, 2002). However, we are interested in recovering the connection between Juan Manuel de Rosas and his officials with certain protagonists, such as Francisco Muñiz.

His figure is well known in Argentina because his name is on one of Buenos Aires’s main hospitals dedicated to infectious diseases, formerly the Casa de Aislamiento (Sánchez Doncell et al., 2023). Muñiz was born in Monte Grande, Buenos Aires province, in 1775 and participated in one of the battalions that repelled the English invasions. He studied at the aforementioned Medical Institute and he acted as a surgeon in various military campaigns, also serving as a doctor in Luján and Chascomús. In 1824, and for decades, he was a member of the Medical Tribunal. In 1827, and for a very short time, he was given access to the subject of “Theory and practice of childbirth, illnesses of children and newly-born women,” a chair at the University of Buenos Aires, from where he was removed because he was not a doctor, a title he only obtained in 1844. He is included in the generation of independence, both for his new ideas and his participation in the Patriotic Literary Society and to the humanitarian care of the wounded, with extensive experience on the battlefield (Sarmiento, 1885).

Muñiz represents the archetype of the nineteenth-century scholar, with interests in very diverse fields. He explored natural sciences in general, medicine, and veterinary medicine. Throughout his life, he displayed an unusual curiosity, as evidenced by his studies of the rhea, an American bird similar to the African ostrich, his descriptions of the *boleadoras*, the weapons of the gauchos, and his collection and study of the bones of extinct mammals, donated to European museums and academies. The reason for this and similar questions was an exchange of letters with Charles Darwin in 1847 (Sarmiento, 1885). Such studies were rare in the country, which lacked naturalistic studies. He possibly knew or had as a teacher Amadeo Bonpland, who accompanied Alexander von Humboldt on his travels. Bonpland taught at the Institute and the University of Buenos Aires in 1820 (Babini, 1949). Muñiz, interested in a wide range of subjects, was self-taught and willing to take risks, observe, and repeat what had been done in other contexts. He also relied on experience and his training and practice as a surgeon, which was less bound by theories and authoritarian criteria.

In 1838, financial difficulties led the Rosas administration to implement severe adjustments to the entire educational and healthcare system. As a result, the Casa de Niños Expósitos was left without funding, and a similar situation occurred at the University of Buenos Aires, which lost three of its few doctors (Di Pasquale, 2018). Also, Muñiz, who was living in the Buenos Aires countryside at the time, had to begin replicating Jenner’s test. He knew about it through Segurola or through those who taught at the Instituto Médico, since it had been widely publicized in various Buenos Aires media since the late eighteenth century.

Recognizing that he was encountering an unusual situation, Muñiz composed a letter to John Epps, who was in charge of the Central Vaccine House in London and had served as the medical director of the Royal Jennerian and London Vaccine Institution since 1831. Epps was also an editor of medical journals, including the *London Medical and Surgeon Journal* (Lindsley, 1897, p.240). In this letter to Epps, Muñiz pointed out the difficulties in transporting people and products suitable for vaccination since 1841. He indicated that “the original vaccine, that is, the pustule of the smallpox vaccine unique to our species, has been extracted from one of the animals within the Department” (Sarmiento, 1885, p.41). Muñiz was a surgeon in an area where cattle had populated the fields since the seventeenth century. In 1830, he recognized the virus in an animal without being able to collect it in time because a dust storm prevented him from doing so. When he returned to search for it, the lesion had disappeared. Cowpox appeared on the skin of sick cattle, and since it was not fatal, the animal survived the rash, which lasted a few days. The infection was attributed to the humidity of the soil and the freshness of the grass, or to other internal factors that alleviated the disease. Muñiz found the lesion on a cow on Gualberto Muñoz’s ranch, who, alert to signs of languor and nervousness, may have pointed it out. The scabs, to be effective, had to be removed on the 14th day of infection, and Muñiz used them to vaccinate several children.

The letter to Epps, written in 1842, describes the information with the order, precision, and clarity that Muñiz attributed to a “poor village doctor” (Sarmiento, 1885, p.41). Epps (1805-1869) later devoted himself to phrenology and was a renowned homeopath and passionate advocate of religious reform (Lindsley, 1897). However, his biography little said about his activity as an immunizer, and his contact with Jenner (who died in 1823) may have been due to their connection to a similar educational institution in Scotland. In England and Scotland, legislation prohibited inoculation in 1840, but in 1853, following the impetus of the Epidemiological Society’s Small Pox and Vaccination Committee, vaccination was made mandatory and free of charge (Durbach, 2005).

Thus, Muñiz was associated with an environment with a certain prestige and recognition. In 1842, Epps responded to his letter and thanked him for his contribution and support of the vaccine, applauding the fact that cowpox was also present in Buenos Aires, which had been conclusively confirmed (Sarmiento, 1885). It was strange not only that it had not appeared since it was a common virus found in cattle and that it had not been detected earlier to replace the more cumbersome and unsafe method currently in use. However, this was a period in which descriptions of germ-related diseases, part of the bacteriological paradigm, were not available to physicians, which represented a huge transformation in medical and health training and regulation.

Francisco Muñiz then took the scabs and inserted them with a lancet into his infant daughter, who was only a few months old. He traveled with her to Buenos Aires to vaccinate several other children and then rediscover the “indigenous vaccine.” This offered a glimmer of hope, considering that all the previous paraphernalia had failed. The Medical Tribunal noted the frustrated attempt to obtain the vaccine “arm to arm,” indicating that the scabs were “old and corrupted, similar to two shipments of scabs received from London by the Honorable Minister of Foreign Affairs” (Sarmiento, 1885, p.40).

Recognition for Muñiz was not long in coming. Justo García Valdez, the general manager of vaccination, sent information about the event to London, where he received glowing praise from Manuel Moreno, the minister of the Confederation in England, who was in charge of supplying the scabs to Buenos Aires. The Medical Tribunal also expressed its strong support, considering the doctor’s work commendable for the sacrifices he made in bringing his infant daughter to Buenos Aires and, subsequently, vaccinating other children. In 1844, he obtained his medical degree from the University of Buenos Aires. The same year, the Royal Jennerian Society of London honored him as a member, and he later received distinctions from medical academies in Zaragoza, Barcelona, and Madrid. He was Darwin’s correspondent in 1848. Between 1857 and 1864, he was elected to membership in the academies of Sweden and Norway, and he was awarded other honors, including a knight of the Order of Wasa.

Among these awards and recognitions, he was appointed as a co-judge of the highest provincial authority in 1849, the Medical Tribunal. In 1850, he held the position of professor of childbirth, and in 1854, he became the president of the Medicine School at the University of Buenos Aires. He also participated in the Battle of Cepeda in 1860, where he applied the humanitarian principle of treating the wounded from both sides. Some of his most notable achievements after 1844 included serving as a doctor at the Casa de Niños Expósitos, about which we we found no further information beyond his resignation in 1855 (Sarmiento, 1885). He passed away in 1871 due to his participation in commissions related to the yellow fever epidemic, whose numerous victims prompted a new generation of doctors to initiate important modern hygienic changes, especially in Buenos Aires.

## Viral reservoir and children’s bodies

In all these cases analyzed from the late eighteenth to the mid-nineteenth century, we observed those who transported the virus to “serve” as a biological repository. Human testing was commonplace in the eighteenth century, and even later, prisoners condemned to death were allowed to survive their sentences if they agreed to be guinea pigs in experiments on the application of therapies. However, this is an anomaly that does not appear as such in traditional histories of medicine. On both sides of the Atlantic, it is unabashedly mentioned the use of infants (Babini, 1949; Damiani, 2020; Ramírez Martín, 2022).

One of the best-known cases was from Jenner, also not considered a risky experiment. He wrote the second test of his celebrated pamphlet focusing two children, one of them his son (Jenner, 1802). While in the eighteenth century, it might have been normal to think that a father would use his son or other children in this way, it is curious that in the twenty-first century, we perceive nothing but praise for Jenner and no criticism of his methods (see Riedel, 2005).

Another example of such a situation can be found in the initial account of the arrival at the port of the “true vaccine,” which was delivered by young slaves. This also allows for reflection on the situation of children and other people who were used as carriers for the virus during inoculation. In the case of slaves, we could argue that the absence of any rights over their bodies during this period led to such practices, which required proper nourishment and care for the vaccine’s effectiveness. In contrast to the usual exploitation, it could be considered legitimate at the time, and even beneficial, to use a slave in this way, given that it allowed them to survive a smallpox epidemic.

Between 1803 and 1807, the Spanish crown strove to suppress smallpox epidemics in its American colonies through the Royal Philanthropic Vaccine Expedition, commanded by Francisco de Balmis Berenguer. This famous expedition included 22 orphaned children, each of whom was inoculated with the attenuated virus to provide “arms” upon arrival. Undoubtedly, Balmis’s measure had little medical ethics because healthy children, who had not suffered from the disease, were infected. Thus the “material” in good condition was safe and ensured success. A historiography that values the saving of lives in the Americas considers it the first humanitarian mission in history and the children the unwitting heroes of this odyssey. During the Royal Expedition, it was difficult for even poor mothers to voluntarily give up their children, despite being offered food, clothing, care, and education, so orphanages provided the children under state custody (Botet, 2009; Ramírez Martín, 2022).

Therefore, medical practices on orphans were not unusual nor were they unusual in the Buenos Aires area. In the second half of the nineteenth century, the collection of humanized viruses was reaffirmed at the Orphanage (Asilo de Huérfanos), the name of the former Casa de Niños Expósitos (Díaz, 1876). At this site, infant mortality from various causes, not just smallpox, was much higher than the city average, with a staggering 40% rate between the ages of 0 and 2 (Huidobro, 1881).

To obtain the “fresh” product and inoculate “arm to arm,” vaccinators first recommended that, if the child had parents, they should consult them about previous illnesses. If the information was negative, the child was vaccinated and waited a few days before injecting the child, extracting the virus, and inoculating another adult or child. It was recommended to “choose a robust, healthy child, less than four months old, with pustules six or seven days old from the day of inoculation, with transparent virus without blood or pus” (Amoretti, 1886, p.55). Using the lancet, the vaccinator took the sample and placed it in a preparation between glass plates or tubes, for later use.

The child’s age and overall condition were important because his brief life had allowed him to remain free from tuberculosis and syphilis, diseases that were undetectable before the onset of symptoms, in a pre-bacteriological stage (Porter, 1997). At the same time, if we read carefully, by using viral reservoirs to inoculate children from the asylum, the vaccinators overcame the parents’ concerns and could repeat the procedure several times without confronting families with a potentially cruel decision. As we mentioned before, the three protagonists (O’Gorman, Segurola, and Muñiz) were in charge of the Casa de Niños Expósitos at different times and were responsible for the health and well-being of the young orphans.

Therefore, on many occasions, instead of cultivating viral strains in animals, fresh material obtained from infants, usually orphans, was preferred. Supplying these products, then, required more than the rhetoric of a few doctors who were striving to consolidate vaccination, a difficult project and full of obstacles.

## Final considerations

In Río de la Plata, both colonial and independent authorities disseminated certain immunization practices to combat smallpox epidemics. Jenner’s work and experimentation with survivors provided the foundations and strategies, but the chaotic political situation between the dissolution of the colonial order and the emergence of a new one made the continuation of these measures difficult. An examination of the protagonists reveals that their commitment to certain political causes affected their respective careers, although there are differences in their education background: O’Gorman, was professionally recognized, but was considered an unreliable official because he was believed to be an English spy; Segurola was a priest with varied knowledge; and, finally, Muñiz was a military surgeon interested in the natural sciences. Segurola and Muñiz also practiced medicine officially, although they would not be considered “doctors” in the strict sense. Participation in various armed conflicts, from the British invasions to the wars of independence and civil wars, left a mark on their professional careers, with setbacks, accusations, and general instability.

Smallpox reached dangerous levels among indigenous societies, which remained autonomous until the end of the nineteenth century. Fear of epidemics, known for their high contagion and mortality rates in these communities, prompted inoculations offered by “whites,” bringing virtually hostile societies closer together.

The harsh practices involved in inoculation, especially in young children, and the possibility of contracting serious diseases or even death were not just the stuff of uneducated people. Unsafe inoculation could trigger outbreaks of infection and deprive a once healthy person of life. There is agreement on these aspects in traditional medical history literature, both in that written 50 years ago (Babini, 1949) and in the more recent literature (Botet, 2009; Riedel, 2005; Damiani, 2020; Ramírez Martín, 2022). Despite this, there remains a prevailing trend towards the collective good over the individual, without affecting the basic rationale, which would be the use of human beings as reservoirs. Slaves and minors, especially orphans, were used to maintain and propagate the virus in optimal conditions for inoculations, reflecting a morally reprehensible situation regarding individual rights that were accepted for the benefit of the majority (adults) of the population.^5^

The various names of the products are striking, leaving us uncertain about the precise reference of each. The term “true” vaccine seems to be synonymous with cowpox; an indigenous vaccine rediscovered by Muñiz in cattle in Luján is also noted; and a “degenerate” vaccine refers to the ineffective. Since even the name was unregulated, we have little knowledge of those who sold them, except that a system of preserving the “true” vaccine was maintained, despite many difficulties from official bodies (Protomedicato, Medical Tribunal), but especially from individuals. This deregulation opened up a range of possibilities, allowing practitioners like Segurola to vaccinate in his garden anyone who knocked on the door, alongside other doctors and healers vaccinating their patients in their homes. The material came from scabs brought from abroad and was reserved for use, with a wide variety of results. Those we call “entrepreneurs” were responsible for bringing the scabs from abroad, along with officials and people interested in distributing them among their families and larger groups (army, indigenous people) for reasons that were not always entirely humanitarian: healthy soldiers and allied groups ensured victory in other fields.

This uncertainty about the product also generated a very confusing scenario and cross-accusations regarding the use and quality of the vaccines. All of this allows us to affirm a very artisanal process, whose confidence dwindled in non-epidemic times. Where little scientific interest prevailed, given that, in Buenos Aires, as in the Kent region, cattle were common and grinding animals, so herd owners, but also other people (military personnel, civil servants, and many others) would be aware of illnesses and diseases. There is a long way from knowing exactly what caused cowpox, but it is equally surprising that it was only observed around 1830, and more than 10 years later it was collected. This issue leads us to the poor quality of medical training, as well as the inability to connect veterinary and medical matters, in times of other emergencies and needs, where precariousness was the rule.

The circulation of rumors regarding other diseases, including the resurgence of smallpox, led to resistance to the use of smallpox prevention. Immunization is complex, depending on numerous factors (nutrition levels, stress, age, gender, previous illnesses, and many other issues). At that time, doctors wondered about the variation in reactions among individuals vaccinated, even when their vaccination had been similar. This level of uncertainty related to biological reactions complicates medical claims of benefits.

Trust was built by reducing vaccine-related accidents, as the fear of introducing a foreign substance into the body to combat a future threat diminished once vaccines became more strictly regulated. The danger of contracting smallpox obscured the risk of vaccination, strengthening the “technology of trust,” a system that combines statistics, education, and reliable and safe vaccine production systems (Porras Gallo, Báguena, 2020, p.5).

At the end of the nineteenth century, a state with greater resources promoted mandatory vaccination, presented as the key to the virtual disappearance of smallpox (Di Liscia, 2017). However, control of the disease was not achieved until the public system promoted spaces for systematic and safe production and a vaccine distribution that made it possible to restrict contagion throughout the national territory. This situation is part of twentieth-century Argentine history and a distinct process from the one we attempted to capture in these pages.

## Data Availability

Not deposited in a data repository.
