# Supplementary material for: Instruments and agents of control: smallpox in the Río de la Plata at the beginning of the nineteenth century
Source: Hist Cienc Saude Manguinhos. 2026 Feb 23;33(Suppl 1):e2026001. doi: 10.1590/S0104-59702026000100001en (PMC12928200; doi:10.1590/S0104-59702026000100001en)
Supplement: Translation

# Translation: Instrumentos y agentes de control: la viruela en el Río de la Plata a comienzos del siglo XIX

**DOI:** 10.1590/S0104-59702026000100001

**Authors:** María Silvia Di Liscia

**Affiliations:** i Profesora titular, Instituto de Estudios Históricos y Sociales de La Pampa/Universidad Nacional de La Pampa. Santa Rosa – La Pampa – Argentina silviadiliscia@gmail.com

**Keywords:** Viruela, Río de la Plata, Siglo XIX, Inmunización

## Abstract

Se investigan distintos sistemas para controlar la viruela a principios del siglo XIX, considerando el accionar de diferentes agentes, mecanismos y estrategias intervinientes en su organización, fabricación y distribución. Esta peligrosa enfermedad apareció en América desde la invasión europea. Las prácticas de inmunización llegaron al Virreinato del Río de la Plata en 1805, coordinadas primero por el Protomedicato y luego a través del Conservatorio de Vacunas. Las dificultades económicas y guerras del naciente Estado complicaron la inoculación de virus humanos y cowpox. Se utilizaron seres humanos como reservorio viral, sin lograr controlar las epidemias. La replicación de las experiencias europeas para obtención de virus similares no proporcionó un producto seguro.

## Avatares sobre la viruela y su prevención

La viruela, enfermedad hoy desaparecida, fue durante siglos altamente peligrosa por su alta morbilidad y letalidad en personas carentes de inmunidad, con graves consecuencias en la salud de los supervivientes. El virus *variola* se contagia de hombre a hombre por el aire; la enfermedad inicia con fiebre y una erupción en todo el cuerpo que puede dejar marcas indelebles y en ocasiones también ceguera. A diferencia de otros males, dispuso de una batería de productos para prevención, como la inoculación brazo a brazo y la variolización.^1^ Ambos sistemas, que limitaron la enfermedad en el siglo XIX, eran recursos inseguros, riesgosos e incluso mortales sobre todo en los menores de edad.

El término “vacunación” se consolidó luego de 1881, cuando Louis Pasteur lo utilizó por primera vez para nombrar su vacuna contra la rabia como homenaje a Edward Jenner. Por extensión se utilizó para todos los productos artificialmente producidos que se introducen en el cuerpo de humanos o animales para generar una reacción inmunológica y preventiva frente a una determinada enfermedad (Moulin, 2010). La antivariólica se considera una de las “tecnologías transferibles” que salvó millones de vidas en todo el mundo y permitió la erradicación de la enfermedad (Altenbaugh, 2018, p.4).

La abundante bibliografía sobre la viruela y su relación con la vacunación a nivel global no puede referirse aquí en su totalidad (Holmberg et al., 2017; Blume, 2017), aunque es preciso considerar los aportes más recientes que debaten las relaciones entre la expansión de información sobre prácticas y estrategias de prevención desde finales del siglo XVIII (Few, 2010; Bhattacharya, Brimnes, 2010). En relación al espacio denominado hoy República Argentina hay trabajos actualizados sobre el impacto de la viruela y su control en el territorio nacional (Jiménez, Alioto, 2017; Di Liscia, 2023).

Nos interesa profundizar en las primeras décadas del siglo XIX, cuando se sacude el escenario americano con el proceso general de independencia hasta los intentos de reorganización política de mediados del XIX. Dado que estas prácticas de inmunización fueron casi la única actividad sanitaria pública, el trabajo también se concentra en el perfil de sus protagonistas, como el protomédico Miguel O’Gorman, el deán Saturnino Segurola y el naturalista Francisco Xavier Muñiz. También se presta atención a otros actores vinculados con las prácticas de inoculación, ya fuesen emprendedores que lucraban con ellas, o bien, niños utilizados como reservorios. Se utilizan fuentes documentales, crónicas, informes y registros oficiales y de la prensa de entonces, así como textos editos e inéditos y tesis de médicos contemporáneos.

## Los protagonistas

### Durante la colonia

La mayor parte del actual espacio argentino, formalmente bajo el imperio español, no tuvo el control efectivo de la metrópoli. Multitud de pueblos originarios estaban establecidos y circulaban de norte a sur por un espacio diverso y rico en recursos. Aunque separados por una frontera y en pie de guerra en determinadas épocas, tanto los españoles como sus descendientes criollos, así como mestizos, negros e indios no tenían existencias separadas y se entremezclaban en tiempos de paz (Di Meglio, 2012).

En 1776, se organizó el Virreinato del Río de la Plata. Su capital, el puerto de Buenos Aires, fundado en el siglo XVI, era por entonces un pequeño poblado, limitado al oeste por el territorio de numerosas etnias indígenas. La población se distribuía de manera desigual en un área extensa y mal conectada: Salta, Tucumán y Jujuy en el Norte, la región cuyana, correspondiente a las provincias de Mendoza, San Juan y San Luis y la central, con Córdoba y Santiago del Estero, así como las provincias del Litoral. En esos territorios también ejercían su dominio las órdenes religiosas, relativamente apartadas del poder real. Como en otras áreas, la metrópoli conformó ciudades, gobernadas por Cabildos (Maeder, 2018).

La aparición de la viruela en América se corresponde con el ciclo de conquista y colonización. En todo el continente, la enfermedad tuvo una alta morbi-letalidad entre las poblaciones originarias, dado que su sistema inmunológico carecía de la “memoria” para hacer frente a una patología experimentada y sufrida por los europeos y africanos desde hacía siglos (McNeill, 1976). En el Virreinato, la enfermedad se registró desde el siglo XVII con oleadas en la ciudad de Buenos Aires y posiblemente en el resto del territorio.^2^

Dado que el éxito de la ocupación española tenía como base el uso de indígenas como servidores y trabajadores forzados, los conquistadores y misioneros extendieron la inoculación. Esta práctica se realizó a finales del siglo XVIII de manera sistemática entre los guaraníes de las misiones del nordeste, bajo el imperio de los jesuitas, mucho antes de la llegada de la expedición de Francisco Balmis al continente (Jiménez, Aliotto, 2017). En esas reducciones, se sucedieron mortíferas epidemias de viruela en 1732, 1733, 1738 y 1764 y los curas inocularon a 7.414 indígenas (Penna, 1885). Esa estrategia era la única a mano en tiempos epidémicos y consistía en tomar de un superviviente las costras con virus variólico y colocarlas a otra persona para inmunizarla. Su eficacia era reducida, puesto que los sanos podían desarrollar la enfermedad, aunque de forma más atenuada, además de que, si la práctica no estaba hecha en condiciones higiénicas o el donante tenía enfermedades previas, podía acarrear infecciones o el contagio de otras dolencias.

Durante la colonia, muchas de las competencias sanitarias, entre ellas la vacunación, fue responsabilidad del Real Protomedicato. Surgido en España en el siglo XV, se componía de técnicos encargados de regular el arte de curar con funciones docentes. Como otras instituciones, tuvo su réplica americana desde 1570 en el Virreinato de Nueva España y en el del Perú (Lanning, 1997). Juan José Vértiz, virrey a cargo del Río de La Plata, solicitó en 1779 la creación del Protomedicato, así como la Casa Cuna para niños expósitos (Gutiérrez, 1877; Babini, 1949). Pero la Corona recién formalizó el nombramiento efectivo del protomédico en 1798, y más que por la usual desidia en asuntos burocráticos – queja muy frecuente – fue por la designación del funcionario a cargo.

El encargado del Protomedicato en el Río de la Plata desde 1798 hasta prácticamente su disolución en 1822 fue el médico irlandés Miguel O’Gorman. Formado en París como doctor en medicina, ingresó al servicio español en 1766. Se lo envió a estudiar específicamente el proceso de variolización a Inglaterra, método que luego aplicó con éxito, inoculando a toda la Corte española (Ramírez Martín, 2022). La situación de O’Gorman nunca fue sencilla en Buenos Aires y hasta el monarca, Carlos IV, tenía dudas de su nombramiento como protomédico, dado que se lo suponía un espía inglés (Damiani, 2020).

La calidad y cantidad de servicios médicos se reducía en el caso porteño a escasos hospitales, para varones y mujeres, mantenidos por órdenes religiosas, y en el resto del territorio rioplatense sólo algunos pobres edificios podían llamarse tales, de gran precariedad y con carencias notables (Damiani, 2020). Tampoco sorprende puesto que eran sitios muy alejados de la higiene y donde se atendían solo a los muy pobres; las personas con cierto peculio tenían médicos y abonaban onerosos remedios en boticas y otros centros de provisión de drogas, que también el Protomedicato debía controlar.

En 1799, el Protomedicato estaba conformado por O’Gorman y seis funcionarios más, de los cuales sólo tres disponían de cierta formación médica. Para “precaver el empirismo en el Virreinato” (Gutiérrez, 1877, p.389), una Real Cédula habilitó la creación de una cátedra médica, impartida por O’Gorman y un cirujano (Agustín Fabre), que no tuvo más que nueve estudiantes en 1801. La formación era limitada, dado que los futuros profesionales carecían de un anfiteatro o de laboratorio de química y tenían que adquirir sus propios instrumentos de disección. En 1806 egresaron los primeros profesionales, pobremente capacitados (Gutiérrez, 1877).

En 1803, se mencionaron tres doctores y 29 licenciados habilitados a tal fin, con penalidades para los no habilitados (Gutiérrez, 1877). Las autoridades culpabilizaron a la población de ceder su salud a curanderos y falsos médicos, pero en este período la medicina no tenía asideros científicos y ambos especialistas se diferenciaban más por el ejercicio del poder médico que por las certezas experimentales (Di Liscia, 2002).^3^

Pero, a pesar de todas estas carencias, en Buenos Aires ya estaba la información sobre las nuevas técnicas de inmunización para evitar la viruela, dado que O’Gorman las conocía de primera mano por su viaje y contacto en Inglaterra. A diferencia de la inoculación, donde se introducía el virus vivo de un superviviente de la viruela a uno sano, se trataba aquí de introducir el *cowpox*, que podía hacerse en recipientes o a través de personas. La primera práctica, si bien era posible, no tenía muchos defensores porque el fluido resultante se secaba y no producía efecto alguno. La segunda introducía a personas con determinadas características como portadoras del virus vacuno en su sistema, que lo traspasaban mediante la lanceta a otras. Jenner había destacado incluso con dibujos alusivos cómo debía ser la pústula resultante de la inoculación con las agujas y el tiempo de espera; si eso no se producía la operación se invalidaba (Jenner, 1802).

El inicio de la variolización en el Río de la Plata se debió al arribo de un empresario portugués, Antonio Machado Carvalho, quien llegó a principios de agosto de 1805 en un buque con esclavos inoculados, trayendo la “verdadera vacuna” en “dos negros pequeños” (Díaz, 1876, p.15; Caffarena Barcenilla, 2016). Una vez arribado el barco al puerto de Buenos Aires, O’Gorman llamó a toda la población a la Real Fortaleza, y, entre el 1º y el 4 de agosto, Justo García Valdez y Silvio Gafarat, dos de los vacunadores, inocularon a 34 personas. El 9 de agosto, y a través de varias personas que habían desarrollado la “verdadera vacuna”, el beneficio se extendió a 200 personas (Díaz, 1876; Coni, 1878). Penna (1885) agregó que entre los inoculados estaban los de la Casa de Niños Expósitos.

La anexión del adjetivo “verdadero” al producto extraído de los brazos infantiles se debe a que, en ese momento, existía una prevención frente a otros de eficacia limitada. Por entonces, no se cita ninguna experiencia similar a la de Jenner de reproducción del *cowpox*, lo cual parece curioso, dado que en ambas regiones había personas en contacto con vacunos. La situación puede deberse a la formación médica, orientada a un paradigma galénico, de escasa preocupación por asuntos veterinarios.

O’Gorman redactó en 1805 una instrucción sobre la práctica, que se reprodujo en la imprenta de la Casa de los Niños Expósitos. Y es una rareza, ya que se trata del único que alude a cuestiones médicas de un cúmulo de obras dedicadas a la información burocrática oficial, como edictos y reales cédulas, o guías, textos religiosos e informes (Maeder, 2018). Este documento fue posiblemente el modelo que siguieron otros similares, como el de Saturnino Segurola, quien inició su actuación en 1807. Segurola había nacido en Buenos Aires en 1776, donde murió en 1854. Obtuvo en Chile un doctorado en teología, y desde principios del siglo XIX sus intereses estaban volcados al control de la viruela, dado que introdujo en Santiago la vacuna en 1805. No era médico, pero sí un interesado en diversos asuntos históricos y culturales, reuniendo una colección notable de mapas, documentos oficiales, periódicos impresos, sermones, memorias, manuscritos y objetos donados a museos y bibliotecas, que conservó en repositorios y archivos hasta su muerte y luego se donaron a la actual Biblioteca Nacional.

El Protomedicato, bajo el control de Justo García Valdez, lo responsabilizó en 1807 de la conservación de la vacuna, un hecho que puede parecer poco importante, pero era esencial para la época, debido a que unos meses antes se habían producido las invasiones al territorio rioplatense por fragatas inglesas. El hecho forzó la organización popular de milicias, dado que el virrey abandonó Buenos Aires, episodio que demostró a los criollos los límites del poder imperial, con implicancias en el proceso posterior de la independencia (Goldman, 1998). Segurola desempeñó entonces un papel fundamental al evitar la pérdida del “fluído vacuno” (Real Protomedicato…, 1940).

En 1805 habían ingresado esclavos con el virus en sus cuerpos (supuestamente del *cowpox*). Pero ¿cómo utilizar esa correa de trasmisión inmunológica para otros, a través de las lancetas y de nuevas aplicaciones que pudiesen reservar la “vacuna verdadera”, en términos de la época? El encargado del procedimiento fue Segurola, y seguramente lo hizo con dos métodos a la vez: resguardando las costras seguras en vidrios y a través de niños, dado que estaba encargado de la Casa de Niños Expósitos. Estas hipótesis no pueden refrendarse con fuentes documentales dado que nada dicen de las técnicas, ya fuera por su carácter esotérico o porque eran tan evidentes que no había nada desconocido para narrar.

Pero un año después, Segurola hubo de defenderse de acusaciones sobre el tipo de producto suministrado y el lugar de las aplicaciones:

Quien creyera que unos facultativos sensatos después de pedirme la Bacuna para cumplir con sus personas favoritas y decir públicamente en las casas de este pueblo que yo era el único que conservaba la Bacuna y que si no fuera por mi anhelo se habría extinguido así en este pueblo como en todo el Virreiynato tuviesen el empeño de no concurrir a mi casa porque el líquido mío no servía? (Nota…, 1940, p.76).

Con palabras iracundas, trataba a sus acusadores de envidiosos, de formar un plan ruin, de tener una mente enferma que en vez de pensar en el “interés público”, sólo tenía en mente “sus intereses particulares”. A la vez, invitaba que el Protomedicato

para lavar esta nota de infamia que de algún modo puede ser trascendental a los miembros de este tan respetable cuerpo, no encuentro otro recurso del que V.S se sirva señalar si le parece el día más oportuno convocando a los facultativos de su agrado, para presentar varias personas Bacunadas de mi mano para que reconociéndose la Viruela manifiéstese al público engañado por los medios que indicare la prudencia a V.S. la legitimidad de semejante específico que he administrado en millares de individuos de todas las edades (Nota…, 1940, p.79).

En este documento la vacunación ya está en debate entre lo individual y lo colectivo, pero no por los derechos individuales a negarse a un producto artificial para evitar el contagio colectivo, sino en relación con la tarea que demanda y su beneficio pecuniario. Segurola había vacunado miles de manera gratuita y los que lucraban con la vacuna, afectados en sus honorarios, podrían haber elevado quejas sobre el producto al Protomedicato. La forma de resolver esta cuestión, propuesta por el clérigo, se nos escapa: ¿podía probarse, con las técnicas de entonces, que las personas inmunizadas lo estaban realmente? Lo que sí advertimos es la escasa instrumentación institucional de la medida, que dependía de una persona y de sus medios. La situación intentó modificarse un tanto en el período de gobierno posterior, con relativo éxito.

### Independencia y más allá

En 1810, las noticias sobre la caída de la Junta Central en Sevilla a manos de la invasión francesa en la península le imprimieron una nueva dinámica al escenario rioplatense, donde ya existían sectores afines a promover cambios e incluso a romper con el gobierno metropolitano. Hasta 1820, en todo el espacio hoy argentino, involucrando incluso a las parcialidades indígenas, se desarrolló lo que se denomina a grandes rasgos la “guerra de independencia”. Este conflicto militar empezó con expediciones de la Junta Gubernativa de Buenos Aires – que reemplazó al virrey –, enviadas a distintos puntos del territorio, con un éxito desigual. En años subsiguientes, el panorama de la guerra se complicó incluyendo otros frentes con las provincias del Litoral, la Banda Oriental y con una poderosa ofensiva portuguesa, al mismo tiempo que un ejército cruzaba los Andes para avanzar sobre los realistas desde Chile y Perú (Di Meglio, 2007).

A la vez, se sucedieron toda una serie de cambios en el gobierno de las entonces denominadas Provincias Unidas del Sur entre los cuales merece señalarse la militarización de las élites en un contexto de enormes dificultades socioeconómicas, debidas a la imposibilidad de gestar un orden político nuevo y a las transformaciones de los elencos gubernamentales, así como al complicado panorama internacional (Goldman, 1998). Tales cuestiones sin duda afectaron a todas las instituciones, entre ellas las dedicadas a asuntos médicos, de ya precario desarrollo.

En 1810, a través de un curioso documento, Miguel O’Gorman declaró el traspaso del conservatorio de vacuna a la Junta Gobernativa de las Provincias. También denotaba los esfuerzos realizados desde 1805 para mantener el conservatorio y llevarlo gratuitamente a otros puntos del Virreinato, a través de dos profesores de medicina y un ayudante en la Casa de Niños Expósitos y en el Hospital de Residencia. Pero cinco años después se había abandonado la vacunación y cundía nuevamente la viruela. Las razones aducidas eran que los responsables de proveer y distribuir el producto habían olvidado esa obligación porque no recibían ningún peculio, y en ese entorno, el único continuador había sido Segurola. Se insistía en que había facultativos que expandían nociones contrarias a la vacunación en “personas menos instruidas”, a lo cual la institución había mandado a “desimpresionar al Público”, so pena de multa (Real Protomedicato…, 1940, p.79).

En un clima político enrarecido, las autoridades coloniales habían intentado, sin mucha eficacia, variolizar, y traspasaban ese deber al gobierno independiente. El Protomedicato continuó, aunque bajo el control de Saturnino Segurola, quien mantuvo el preparado en vidrios en un establecimiento *ad ho*c e intentó su propagación entre la población. Se anexó un Instituto Médico en 1813, que disponía de un anfiteatro anatómico, cuya principal función era la incorporación de los futuros médicos a la tarea militar (Gutiérrez, 1877). Para ello, el conocimiento de cirugía era esencial, dado que en los campos de batallas las heridas podían ser fatales sin un cuerpo médico acorde y también para prevenir el contagio de la viruela con la inoculación de los soldados.

En 1811, Segurola detalló el envío entre vidrios de costras entre placas de vidrio, indicando a quien se las había requerido, Celedonio del Castillo, que, para usar dichas costras, debía “triturarlas reduciéndolas con alguna curiosidad a 5 pequeños o menudos polvos, verificado esto se humedecen con algunas gotas de agua común hasta formar una especie de humor graso, que se tomara con la lanceta y se aplicara en el modo ordinario” (Carta…, 1940a, p.82). Este sistema se utilizó hasta finales del siglo XIX, aún con variaciones en el sustrato para ligar las muestras, pero es en general lo que se describe como vacuna seca, que se debía hidratar para ser efectiva. Su efectividad era reducida, sembrando dudas sobre la fiabilidad del proceso en general (Di Liscia, 2023). Celedonio del Castillo era un importante contacto entre Buenos Aires y el Nordeste en las guerras de la independencia. Es probable que el material tuviese como fin vacunar a soldados y precaverlos de la viruela, pero también a indígenas, dado que del Castillo ejercía como gobernador delegado de Apóstoles en Misiones, antiguo espacio vinculado a las reducciones jesuíticas guaraníes (Codeseira del Castillo, 2012).

En 1813, con el apoyo de las autoridades policiales porteñas, Segurola publicó su famoso reglamento, donde se instituía un poder médico difícil de entender, dado que su autor no lo era, y se requerían instrumentos legales difíciles de implementar. Pero se trató de una de las pocas reglamentaciones del gobierno independiente referida a cuestiones sanitarias. Su elaboración se basaba en el de 1805, ajustando la recepción, obligatoriedad y difusión de la vacunación: por ejemplo, se solicitaba a los alcaldes de barrio, funcionarios existentes en la colonia y que persistieron hasta 1820, realizar un padrón de vacunados y no vacunados, y les otorgaba función de policía, como “en Europa”. Las personas debían presentar una papeleta con la certeza de haber recibido el producto, so pena de multa. El reclamo era más exigente para las familias con niños pequeños y los comandantes de distintos cuerpos del ejército (Reglamento…, 1940).

El reglamento denotaba la intencionalidad de trasladar el fluido vacunal más allá de Buenos Aires, dejando entrever las dificultades, toda vez que no se disponía de un espacio público donde conservar y colocar la vacuna: “Que para la dicha operación concurrirán como hasta aquí a la casa de mi morada, hasta tanto que el Sr. Juez de Policía arbitre con el Exmo. Cavildo una Casa en el medio del Pueblo donde con más comodidad puedan concurrir los interesados” (Carta…, 1940b, p.87). Esa situación no se produjo hasta avanzado el siglo XIX. Tanto por desidia y problemas de organización como por dificultades políticas, Segurola siguió colocando la vacuna casi de manera particular en su morada de la calle Puán, eso lo reconocieron posteriormente sus biografías (La personalidad…, 1940).

El deán atendió también a otros requerimientos, solicitados por su núcleo político, como el realizado por Manuel Belgrano para el ministro plenipotenciario de los Estados Unidos ante Brasil, Thomas Sunter, quien deseaba “hacer experiencias por sí con la vacuna verdadera” (Carta…, 1940c, p.88). En Río de Janeiro en 1815, dónde Belgrano se encontraba por otros asuntos, Sunter solicitó el producto aduciendo que el conocimiento minucioso de Segurola había trascendido las fronteras rioplatenses. E indicó “que los médicos aseguran que degenera muy prontamente en viruelas” (p.88). Belgrano, comprometido con el conocimiento científico, veía un error en tal afirmación, y requería a Segurola el envío del material a fin de probar lo contrario. Pero tal “degeneración” sin duda existía, dado que el virus no tenía posibilidad de subsistir sin determinadas condiciones de temperatura y humedad y si se inoculaba con virus vivos, se podía contagiar la enfermedad a quienes no la tenían.

Casi a vuelta de correo, el deán envió a Brasil un paquete de vacunas, e indicó con cierta altanería que lo había hecho en más de una oportunidad tanto a ingleses como a portugueses, quienes no tenían en cuenta de que la vacuna podía contagiar la viruela. Ese descuido no había sucedido en Buenos Aires gracias a su intervención: “Son innumerables las veces que he remitido la Vacuna a esa así para ingleses como para los naturales del país, pero según tengo entendido apenas ha servido … de que nace que no queriendo poner los medios para su conservar, suele perderse la Vacuna con este motivo echar mano de la Viruela natural y asegurar que la Vacuna degenera” (Carta…, 1940d, p.89).

Segurola entregó el material gratuitamente a Sunter, quien le agradeció profusamente (Carta…, 1940e, p.90), expresando la eficacia de la vacuna, probada en uno de sus hijos (“experimenté su pureza en uno de mis niños”).^4^ Comentó que saldría en defensa de Segurola, indicando a los médicos de Río de Janeiro la calidad del producto: “Hice lo mismo que el Doctor Jenner y … es un honor haber hecho avanzar la utilidad de su noble descubrimiento, que usted ha protegido tan desinteresadamente durante tantos años” (Carta…, 1940e, p.91).

En años subsiguientes, Segurola continuó recibiendo requerimientos de vacunas de distintos puntos tanto para distribuir en las familias de sus conocidos como entre la población en general. Entre 1816 y 1821, Juan Martín de Pueyrredón solicitó la vacuna para Córdoba (Carta..., 1940f); Dámaso Antonio Larrañaga para Montevideo y la Banda Oriental (Carta…, 1940g) y Tomás Godoy Cruz, para Mendoza. En este último caso, se indicaba la resistencia a la inoculación de la población con productos ineficaces, traídos desde Chile (Carta…, 1940h).

Por entonces comenzaba otro proceso en Buenos Aires, a través del liderazgo de una élite apartada de la centralización que inició una serie de políticas liberales y autónomas. Entre ellas, se suprimieron los cabildos y sus funcionarios, produciéndose un avance de la frontera bonaerense (Ternavassio, 1998). En 1822, en esa misma estela de renovación, el Protomedicato se reemplazó por un Tribunal de Medicina (Sarmiento, 1885). Bajo la égida de Bernardino Rivadavia, surgieron una serie de instituciones claves vinculadas a la situación médico-sanitaria como la Sociedad de Beneficencia, encargada de hospicios y hospitales; la Academia de Medicina, entre otras de artes y ciencia y la Universidad en Buenos Aires en 1821, que incorporó el Instituto Médico anteriormente creado, aunque con cinco cátedras solamente (Gutiérrez, 1877).

Dado que Segurola continuaba aun siendo conservador de la vacuna, se le ordenó el envío de “virus vacuno” para el fuerte de Carmen de Patagones (Orden…, 1940). Esta disposición y otras similares dan cuenta del interés gubernamental por instrumentar la obligatoriedad de la vacunación, indicándose medidas al respecto con una circular dirigida a los cirujanos acantonados en los fuertes de la frontera: Guardia del Monte, Chascomús, Magdalena, San Vicente, Luján, Pilar, Capilla del Señor, Cañada y Areco. El sistema, esbozado décadas antes, se reorganizó con administradores de vacuna y otros funcionarios que recibían un estipendio de 45 pesos por mes y debían inscribir a los vacunados en un registro. Incluso se suministraría transporte a través de caballos de los maestros de postas (Penna, 1885).

Segurola tenía a su cargo seis vacunadores más en esta tarea, refrendada por una publicación oficial, que en 1822 dio a conocer un informe de Jenner donde se indicaba cómo y dónde vacunar, la forma en que se producía la roncha y otras precisiones, aunque nada se dice sobre el proceso de obtención del producto. No sabemos la cantidad de personas vacunadas, pero quizás la calidad del producto no sería la deseada, dado que la viruela retornó en 1825 (Penna, 1885).

Hasta 1829, Segurola desempeñó tareas muy diversas además de las descriptas, tanto de índole cultural como social y política: fue diputado en 1812, director de la Biblioteca Pública y encargado de la Casa de Niños Expósitos (Dr. Deán Saturnino..., 1940). Con el ingreso de Juan Manuel de Rosas en el escenario provincial bonaerense, muchas de estas actividades culminarían, y en especial esta última, dado que la atención de los huérfanos se interrumpió en 1838 por razones presupuestarias.

Rosas fue gobernador de 1829 hasta 1852, cuando fue vencido por una coalición, iniciándose otro período en que la historiografía enmarca el inicio del orden nacional. El territorio pasó a denominarse la Confederación Argentina, con provincias aliadas o en pugna, que mantuvieron cierta autonomía. Durante su gobierno, desplegó una política de alianzas con determinadas parcialidades de indígenas (los “indios amigos”), que se encontraban en Tapalqué y Azul, cerca de los fuertes y recibían raciones, incorporándose a su vez en batallones para derrotar a otros grupos étnicos y extender la frontera bonaerense. Hacia 1830, avanzó en la campaña una epidemia de viruela. El cónsul inglés en el Río de la Plata, Woodbine Parish (1958), expresó que, frente al sufrimiento de los indígenas aliados a Rosas, éste logró convencerlos de inocularse el virus para estar a salvo. Sobre estos aspectos que implicarían la complejidad de las relaciones diplomáticas tejidas entre grupos supuestamente enfrentados, frente a la realidad de las alianzas entre indígenas y blancos, no vamos a volver aquí, ya que los hemos examinado en otros trabajos (ver Di Liscia, 2002). Pero sí nos interesa recuperar la vinculación de Juan Manuel de Rosas y sus funcionarios con determinados protagonistas, como Francisco Muñiz.

Su figura es muy conocida en Argentina, debido a que su nombre está en uno de los principales hospitales porteños dedicado a enfermedades infecciosas, anteriormente la Casa de Aislamiento (Sánchez Doncell et al., 2023). Muñiz nació en Monte Grande, provincia de Buenos Aires, en 1775 y participó en uno de los batallones que repelió las invasiones inglesas. Estudió en el Instituto Médico anteriormente nombrado y con esa formación actuó como cirujano en diversas campañas militares, desempeñándose también como médico en Luján y Chascomús. En 1824, y durante décadas, fue miembro del Tribunal de Medicina; en 1827, y por muy poco tiempo, accedió a la asignatura de “Teoría y práctica de partos, enfermedades de niños y recién paridas”, una cátedra en la Universidad de Buenos Aires, de donde se lo desplazó porque no era doctor, título que obtuvo recién en 1844. Se lo incluye en la generación de la independencia, tanto por sus ideas nuevas como por su participación en la Sociedad Patriótica Literaria como respecto al cuidado humanitario de los heridos, con una amplia experiencia en el campo de batalla (Sarmiento, 1885).

Muñiz representa el estereotipo del sabio decimonónico, con intereses en campos muy diversos: incursionó en las ciencias naturales en general, la medicina y la veterinaria. Manifestó a lo largo de su existencia una curiosidad fuera de lo común, como lo atestiguan sus estudios sobre el ñandú, un ave americana similar al avestruz africano, las descripciones de las boleadoras, armas de los gauchos, y la recolección y estudio de huesos de mamíferos extintos, donados a museos y academias europeos. Motivo de tal cuestión y de otras similares fue un intercambio epistolar con Charles Darwin en 1847 (Sarmiento, 1885). Tal formación era poco frecuente en el país, que carecía de capacitación en estudios naturalistas. Posiblemente conoció o tuvo como profesor a Amadeo Bonpland, acompañante de Alexander Von Humboldt en sus viajes. Bonpland enseñó en el Instituto y la Universidad de Buenos Aires en 1820 (Babini, 1949). Muñiz, interesado en temas muy diversos, era autodidacta, y estaba dispuesto a arriesgarse, observar y repetir lo que se había realizado en otros contextos. Además, obedecía a la experiencia y a su formación y práctica como cirujano, menos ceñida a teorías y criterios de autoridades.

En 1838, las dificultades presupuestarias llevaron a que el gobierno rosista realizara un severo ajuste en toda la estructura educativa y sanitaria. La Casa de Niños Expósitos quedó sin financiamiento y similar situación acaeció en la Universidad de Buenos Aires, que perdió tres de los pocos médicos que tenía (Di Pasquale, 2018). Por esas fechas, Muñiz, que residía por entonces en la campaña bonaerense, debió iniciar la réplica de la prueba de Jenner, de la cual había tenido noticias a través de Segurola o de quienes dictaban las cátedras en el Instituto Médico, puesto que había sido muy publicitada en distintos medios porteños desde finales del siglo XVIII.

Muñiz, consciente de que estaba frente a una situación inusual, envió una carta a John Epps, por entonces a cargo de la Casa Central de la Vacuna en Londres y *medical director*, desde 1831, de la Royal Jennerian and London Vaccine Institution, además de editor de revistas médicas como el *London Medical and Surgeon Journal* (Lindsley, 1897, p.240). En esa misiva dirigida a Epps, Muñiz indicaba las dificultades de traslado de personas y productos aptos para vacunar desde 1841. De manera pormenorizada, indicó la obtención de “la vacuna original, o sea la pústula de la vacuna privativa de la viruela de nuestra especie, ha sido extraída de uno de los animales dentro del Departamento” (Sarmiento, 1885, p.41). Muñiz era cirujano en un espacio donde el ganado vacuno poblaba los campos desde el siglo XVII. En 1830 había reconocido el virus en un animal sin poderlo recolectar a tiempo, porque una tormenta de polvo se lo impidió y al volver a buscarlo, ya no estaba la lesión. El *cowpox* aparecía en la piel del ganado enfermo y como no era mortal, el animal sobrevivía a la erupción que duraba unos pocos días. La infección se atribuía a la humedad del terreno y frescura de la hierba, o a otros factores internos que permitían aliviar la enfermedad. Muñiz encontró la lesión en una vaca de la hacienda de Gualberto Muñoz, quien, atento a las señales de languidez y nerviosismo, puede habérsela señalado. Las costras, para ser efectivas, debían extraerse el día 14 de la infección, y con ellas, Muñiz vacunó a varios niños.

La carta a Epps, escrita en 1842, describe la información con orden, precisión y claridad que Muñiz dice que pertenecen a un “pobre médico de la aldea” (Sarmiento, 1885, p.41). Epps (1805-1869) se volcó en años subsiguientes a la frenología y fue un reconocido homeópata, además de un ardiente defensor de reformas religiosas (Lindsley, 1897). En su biografía, sin embargo, poco se dice de esta actividad como encargado de la vacunación, y su contacto con Jenner (muerto en 1823) quizás obedeció a que compartieron similar institución de enseñanza en Escocia. En Inglaterra y Escocia, una legislación prohibió en 1840 la inoculación, pero en 1853, a raíz del impulso de la Epidemiological Society’s Small Pox and Vaccination Comitee, se impulsó la obligatoriedad y gratuidad de la vacunación (Durbach, 2005).

Por lo tanto, Muñiz se vinculó a un entorno con cierto prestigio y reconocimiento. Epps contestó en 1842 a su misiva y le agradeció su aporte como activo amigo de la vacuna, aplaudiendo que el *cowpox* también estuviese en Buenos Aires y que se hubiese corroborado fehacientemente (Sarmiento, 1885). Desde luego, era extraño no sólo que no apareciera, siendo un virus común en el ganado vacuno, sino que no se hubiese detectado antes para usarse en vez del sistema utilizado, más engorroso e inseguro. Pero se trata de una etapa donde no estaban a disposición de los médicos las descripciones sobre enfermedades vinculadas a gérmenes, parte del paradigma bacteriológico, que supuso una enorme transformación en la formación y regulación médico-sanitaria.

Francisco Muñiz tomó entonces las costras, las introdujo con una lanceta en su hija de pocos meses y viajó con ella hacia la ciudad de Buenos Aires, para a su vez vacunar a varios niños y así, redescubrir la “vacuna indígena”. Tal cuestión ponía una nota de esperanza dado que toda la parafernalia anterior había fracasado: el Tribunal de Medicina había indicado el intento frustrado de conseguir la vacuna “brazo a brazo” pero las costras estaban “viejas y desvirtuadas, sucediendo lo mismo con dos remesas de costras recibidas de Londres por el Exmo. Ministro de Relaciones Exteriores” (Sarmiento, 1885, p.40).

El reconocimiento para Muñiz no tardó en llegar. Justo García Valdez, encargado general de la vacunación, envió información del hecho a Londres y recibió los encendidos elogios de Manuel Moreno, ministro de la Confederación en Inglaterra, quien estaba encargado de proveer las costras a Buenos Aires. El Tribunal de Medicina también emitió su apoyo elocuentemente, considerando la tarea del médico como loable por los sacrificios realizados al llevar a su hija de meses a Buenos Aires y a partir de ella, vacunar a otros niños. En 1844, obtuvo su diploma como doctor en la Universidad de Buenos Aires; ese mismo año la Real Sociedad Jenneriana de Londres lo designó miembro honorario y luego, recibió las distinciones de academias médicas en Zaragoza, Barcelona y Madrid. Recordemos que fue corresponsal de Darwin en 1848. Entre 1857-1864, las academias de Suecia y Noruega lo escogieron como miembro y se le otorgaron otros honores, como caballero de la Orden de Wasa.

En esa catarata de premios y reconocimientos consta también que, en 1849, se lo designó con-juez del máximo organismo provincial, el Tribunal de Medicina; en 1850, profesor de partos; y, en 1854, presidente de la Facultad de Medicina de la Universidad de Buenos Aires. También participó de la batalla de Cepeda en 1860, donde aplicó la normativa humanitaria de curar a los heridos de ambos bandos. En el rosario de sus logros más notables a partir de 1844, está el de haber sido médico de la Casa de Niños Expósitos, de lo cual no tenemos más datos que su renuncia en 1855 (Sarmiento, 1885). Su muerte en 1871 obedeció a la participación en comisiones vinculadas a la atención en la epidemia de la fiebre amarilla, cuyas numerosas víctimas provocaron que una nueva generación médica iniciara importantes cambios higiénicos modernos, sobre todo en Buenos Aires.

## Reservorio viral y cuerpos infantiles

En todos estos casos analizados desde finales del siglo XVIII hasta mediados del XIX, aparecen a contraluz quienes trasladaron el virus para “servir” de depósito biológico. El ensayo en seres humanos era algo habitual para el siglo XVIII, e incluso posteriormente se permitió a presos condenados a muerte sobrevivir a la pena si aceptaban ser conejillos en experimentos sobre la aplicación de terapias. Pero es una anomalía que no aparece como tal en las historias tradicionales de la medicina, que, a uno y otro lado del Atlántico, indican sin rubor el uso de infantes (Babini, 1949; Damiani, 2020; Ramírez Martín, 2022).

Uno de los casos más conocidos, tampoco valorado como un experimento riesgoso, fue el detallado por el mismo Jenner (1802), al escribir la segunda prueba de su celebrado opúsculo sobre dos niños, uno de ellos su hijo. Si bien en el siglo XVIII podría ser normal pensar que un padre utilizaba a su hijo o a otros niños de esta manera, resulta curioso que en el XXI no se perciban más que alabanzas a Jenner y ningún reparo a sus métodos (ver Riedel, 2005).

Otro ejemplo de tal situación está en el relato inicial de la llegada al puerto de la “vacuna verdadera”, a través de pequeños esclavos, lo que permite también reflexionar sobre la situación particular de los niños y otras personas a quienes se utilizaba como reservorios del virus para inocular. En el caso de los esclavos, bien podría aducirse que, en este período, carecer de cualquier derecho sobre el cuerpo daba pie para este uso que, a su vez, requería alimentación y otros cuidados para su rendimiento. Frente a la explotación usual, podría considerarse en ese momento que era legítimo utilizar de esta manera a un esclavo y aún beneficioso, dado que le permitía sobrevivir en una epidemia de viruela.

Entre 1803-1807, la corona española se esforzó en suprimir las epidemias de viruela en sus colonias americanas a través de la Real Expedición Filantrópica de la Vacuna comandada por Francisco de Balmis Berenguer. En esta famosa expedición iban 22 niños huérfanos, a quienes se les inoculaba sucesivamente el virus atenuado para disponer de “brazos” a la llegada. Indudablemente, la medida de Balmis tenía poco de ética médica porque se contagiaba a pequeños sanos, que no habían padecido el mal, para salvaguardar así el “material” en condiciones y tener éxito. Pero una historiografía que valora el ahorro en vidas en América la considera la primera misión humanitaria de la historia y a los niños, héroes involuntarios de esta odisea. En la Real Expedición, fue difícil que aún las madres pobres entregaran voluntariamente a sus hijos, pese a que se les ofrecía alimentación, vestidos, cuidado y educación, por lo que los orfanatos suministraron los niños bajo custodia estatal (Botet, 2009; Ramírez Martín, 2022).

Las prácticas médicas en huérfanos, por lo tanto, no eran extrañas y tampoco lo fueron en el entorno porteño. En la segunda mitad del siglo XIX, se reafirmaba la recogida de virus humanizado en el Asilo de Huérfanos, nombre de la antigua Casa de Niños Expósitos (Díaz, 1876). En este sitio, la mortalidad de los infantes por diferentes causas, no sólo viruela, era mucho más elevada que la media de toda la ciudad, con una tasa escalofriante del 40% entre 0 y 2 años (Huidobro, 1881).

Para obtener el producto “fresco” e inocular “brazo a brazo”, los vacunadores recomendaban primero que, si el menor contaba con padres, consultarles sobre sus enfermedades previas. Si la información era negativa, se procedía a vacunarlo y esperar unos días para proceder a punzarlo, extraer el virus e inocular a otro adulto o niño. Se recomendaba “elegir un niño robusto y sano, de menos de cuatro meses, con pústulas de seis o siete días a contar desde el día de la inoculación, con virus transparente sin sangre ni pus” (Amoretti, 1886, p.55). Con la lanceta, el vacunador tomaba la muestra y colocaba en un preparado entre placas o tubos de vidrio, para luego utilizarlo.

La edad y estado general del pequeño eran importantes porque su corta vida le había permitido estar libre de tuberculosis o sífilis, enfermedades indetectables antes de la aparición de síntomas, en una etapa pre-bacteriológica (Porter, 1997). Y a la vez, si leemos atentamente, al utilizar como reservorios virales para inocular a niños del asilo, los vacunadores dejaban de lado los reparos de los padres y podían repetir la operación varias veces, porque no iban a tener que hacer frente a la familia ante una medida que podía ser cruenta. Recordemos que los tres protagonistas (O’Gorman, Segurola y Muñiz) estuvieron a cargo en diferentes períodos de la Casa de Niños Expósitos y eran responsables de la salud y bienestar de los pequeños huérfanos.

Por lo tanto, en muchas ocasiones, en vez del cultivo de cepas virales en animales, se prefirió el material fresco obtenido de infantes, generalmente huérfanos. El suministro de productos requirió, entonces, más que el discurso de algunos médicos que se esforzaban por afianzar la vacunación, un proyecto difícil en sí mismo y con no pocos escollos.

## Consideraciones finales

En el Río de la Plata, tanto las autoridades coloniales y las de la etapa independiente difundieron determinadas prácticas de inmunización frente a las epidemias de viruela. La obra de Jenner y la experimentación con los supervivientes proporcionaron las bases y estrategias, pero la caótica situación política, entre la disolución del orden colonial y el surgimiento de uno nuevo, dificultó la continuidad de las medidas. En un examen de los protagonistas, se observa que el compromiso con determinadas causas políticas afectó las respectivas carreras, aunque exista entre ellos diferencias de formación: O’Gorman, reconocido a nivel profesional, pero considerado poco confiable como funcionario en tanto posible espía inglés; Segurola, sacerdote, con variados conocimientos y, por último, Muñiz, cirujano militar e interesado en las ciencias naturales. Los dos últimos también ejercieron la medicina de manera oficial, aunque no serían considerados como “médicos” en sentido estricto. La participación en diversos conflictos armados, desde las invasiones inglesas, las guerras de la independencia y las civiles, dejó una señal en sus carreras profesionales, con sobresaltos, acusaciones y en general, inestabilidad.

La viruela registró peligrosos niveles entre las sociedades originarias, que hasta finales del siglo XIX permanecieron con cierto grado de autonomía. El temor a epidemias, conocidas por su alto contagio y letalidad en esas comunidades, impulsó la inoculación ofrecida por los “blancos” y acercó así sociedades virtualmente enemigas.

Las prácticas cruentas involucradas en la inoculación, sobre todo en niños pequeños y la posibilidad de contagio de enfermedades graves o de la muerte no eran sólo parte de la imaginación de personas sin instrucción. La colocación sin condiciones de antisepsia podía iniciar focos de infecciones y llevarse entonces una vida que, antes del proceso, estaba sana y robusta. Sobre estos aspectos hay acuerdo en la bibliografía tradicional de historia de la medicina, tanto la escrita hace 50 años (Babini, 1949) como la más reciente (Botet, 2009; Riedel, 2005; Damiani, 2020; Ramírez Martín, 2022). Pero la corriente general es el bien colectivo por sobre el individual, sin que se afecte el razonamiento básico, que es el uso de seres humanos como reservorios. Los esclavos y menores de edad, en especial huérfanos, se utilizaron para mantener y reproducir el virus en condiciones óptimas para inocular a otros, situación moralmente reprobable sobre sus efectos individuales, que se aceptaba en pos de las ventajas para la mayoría (adulta) de la población.^5^

Los diferentes nombres del producto llaman la atención y no siempre estamos seguros de cuál es exactamente la referencia de la fuente: vacuna segura y denominada “verdadera”, parece ser sinónimo de *cowpox*; vacuna indígena, aquella redescubierta por Muñiz en vacunos en Luján. La vacuna “degenerada”, aquella que no hacía efectos. Al no estar regulado ni el nombre, tampoco sabemos quién las expendía, salvo que se mantuvo un sistema de conservación de la vacuna “verdadera”, aún con muchas dificultades a partir de los organismos oficiales (Protomedicato, Tribunal de Medicina) pero sobre todo de particulares. Aflora en esa desregulación un abanico de posibilidades: desde Segurola, vacunando en el jardín de su casa a quienes golpeaban la puerta, a otros médicos y curadores haciéndolo a sus pacientes, en sus casas. El material provenía de costras que venían del exterior, y se reservaban para utilizarse, con resultados muy variados. Lo que llamamos “emprendedores” eran aquellos que las traían del exterior, y también, funcionarios e interesados en que circulara entre sus familias y colectivos más amplios (ejército, indígenas) por razones no siempre totalmente humanitarias: soldados y grupos aliados sanos aseguraban la victoria en otros campos.

Pero sin duda, esa indeterminación sobre el producto generaba un escenario también muy confuso y acusaciones cruzadas en relación con el uso y calidad de las vacunas. Todo esto nos permite afirmar un proceso muy artesanal, cuya confianza se adelgazaba en tiempos no epidémicos. Y donde primaba un escaso interés científico, dado que, en Buenos Aires, tal como en la zona de Kent, los vacunos eran animales corrientes y molientes por lo que dueños de rodeos, pero también otras personas (militares, funcionarios y muchos más) estarían al tanto de enfermedades y dolencias. Por supuesto que de allí a saber exactamente qué producía el *cowpox* hay una distancia, pero sorprende igualmente que recién hacia 1830 se observó y más de 10 años después se recolectó. Tal cuestión nos lleva a la escasa calidad de la formación médica, así como a la incapacidad de relación de asuntos veterinarios y médicos, en tiempos de otras urgencias y necesidades, donde la precariedad era la regla.

La circulación de rumores sobre el contagio de otras enfermedades e incluso, la aparición de la viruela, producían rechazo frente al recurso de su prevención. La inmunización resulta compleja, ya que depende de una multitud de causas (niveles de nutrición, stress, edad, género, dolencias previas y muchas cuestiones más). Por entonces, los médicos se preguntaban sobre la variación de las reacciones en los vacunados, cuando habían actuado de similar manera. Ese nivel de indeterminación relacionado con las reacciones biológicas complicaba las proclamas médicas en pos de las ventajas.

La generación de la confianza se logró limitando los accidentes con vacunas, dado que el temor ante la introducción de un fluido extraño en el cuerpo para combatir una amenaza futura desapareció, porque se controlaron los productos a vacunar. Y el peligro a contagiarse de viruela ocluyó el de la aplicación de la vacuna, fortaleciendo la “tecnología de la confianza”, un conjunto que une estadística, educación y sistemas fiables y seguros de producción de vacunas (Porras Gallo, Báguena, 2020, p.5).

Ya a finales del siglo XIX, un Estado con mayores recursos impulsó la obligatoriedad de la vacunación, presentada como la clave de la virtual desaparición de la viruela (Di Liscia, 2017). Pero a su vez, el control de la enfermedad no se logró hasta que el sistema público impulsara, por un lado, espacios de producción sistemática y segura, y por otro, una distribución de vacunas que permitiese restringir el contagio en todo el territorio nacional, situación que es parte de la historia argentina del siglo XX y un proceso diferente al que intentamos plasmar en estas páginas.

## Data Availability

No están en repositorio.

## NOTAS

1 Se denomina “variolización” a una práctica muy antigua, datada desde el siglo XII en China, por la cual se introducían las costras o pústulas de un superviviente de viruela en una persona sana, ya fuera a través de aspiraciones por la nariz como de una lanceta por la piel. El método se expandió en Europa en el siglo XVIII, hasta que las experiencias de Edward Jenner en Gran Bretaña introdujeron el uso de productos con *cowpox*, similar al de la viruela humana obtenido de vacunos, sin que estuviese en claro aún por entonces la relación entre inmunidad y vacunación. La variolización es entonces la inoculación del virus a sujetos sensibles, introduciendo en ellos una infección menos grave que la producida por vía respiratoria, pero a diferencia de la vacunación, podía transmitir la viruela a personas no protegidas. Durante las epidemias, o por escasez de vacunas, se utilizaba como último recurso (L’Eradication…, 1980).

2 Según Besio Moreno (citado en Fiquepron, 2020, p.174), hubo epidemias en Buenos Aires en 1605, 1620-1621, 1627, 1687, 1705, 1717, 1734, 1742, 1778-1779, 1793-1796, 1801-1802, 1829, 1836-1837, 1853, 1862-1869, 1871, 1875, 1879-1880, 1883, 1885 y 1890.

3 Para la medicina oficial, persistía el modelo de explicación humoral sobre la existencia de sustancias corporales (bilis, sangre), cuyo desequilibrio hacía posible la enfermedad. Por entonces se desplegaba otro paradigma, surgido de la observación anatomoclínica, donde las enfermedades provenían de lesiones. Posteriormente, las teorías sobre el germen proporcionaron otro marco explicativo (Porter, 1997).

4 En esta y en las demás citas de textos en otros idiomas, la traducción es libre.

5 Una excepción es Morente Parra (2022), quien problematiza el relato aduciendo a los efectos éticos y su significado.
